# Unveiling human biomechanics: insights into lower limb responses to disturbances that can trigger a fall

**DOI:** 10.3389/frobt.2024.1367474

**Published:** 2024-09-10

**Authors:** Nuno Ferrete Ribeiro, Miguel Armada, João Nunes, Óscar Carvalho, Cristina P. Santos

**Affiliations:** ^1^ Center for MicroElectroMechanical Systems, University of Minho, Guimarães, Portugal; ^2^ LABBELS—Associate Laboratory, University of Minho, Braga, Portugal; ^3^ LABBELS—Associate Laboratory, University of Minho, Guimarães, Portugal

**Keywords:** biomechanical response, slip-like perturbation, fall prevention, target specification, wearable robotic device

## Abstract

**Introduction:**

Slip-related falls are a significant concern, particularly for vulnerable populations such as the elderly and individuals with gait disorders, necessitating effective preventive measures. This manuscript presents a biomechanical study of how the lower limbs react to perturbations that can trigger a slip-like fall, with the ultimate goal of identifying target specifications for developing a wearable robotic system for slip-like fall prevention.

**Methods:**

Our analysis provides a comprehensive understanding of the natural human biomechanical response to slip perturbations in both slipping and trailing legs, by innovatively collecting parameters from both the sagittal and frontal plane since both play pivotal roles in maintaining stability and preventing falls and thus provide new insights to fall prevention. We investigated various external factors, including gait speed, surface inclination, slipping foot, and perturbation intensity, while collecting diverse data sets encompassing kinematic, spatiotemporal parameters, electromyographic data, as well as torque, range of motion, rotations per minute, detection, and actuation times.

**Results:**

The biomechanical response to slip-like perturbations by the hips, knees, and ankles of the slipping leg was characterized by extension, flexion, and plantarflexion moments, respectively. In the trailing leg, responses included hip flexion, knee extension, and ankle plantarflexion. Additionally, these responses were influenced by gait speed, surface inclination, and perturbation intensity. Our study identified target range of motion parameters of 85.19°, 106.34°, and 95.23° for the hips, knees, and ankles, respectively. Furthermore, rotations per minute values ranged from 17.85 to 51.10 for the hip, 21.73 to 63.80 for the knee, and 17.52 to 57.14 for the ankle joints. Finally, flexion/extension torque values were estimated as −3.05 to 3.22 Nm/kg for the hip, −1.70 to 2.34 Nm/kg for the knee, and −2.21 to 0.90 Nm/kg for the ankle joints.

**Discussion:**

This study contributes valuable insights into the biomechanical aspects of slip-like fall prevention and informs the development of wearable robotic systems to enhance safety in vulnerable populations.

## 1 Introduction

Slips are a prevalent and concerning issue, particularly among the elderly population (Chang et al., 2016). For older adults, consequent fractures, especially hip fractures, can be life-altering events, leading to high expenses per episode of care (Ferris et al., 2022). Falls often lead to hospitalization, surgery, and a prolonged recovery process, sometimes resulting in a decline in overall health and mobility. The physical and emotional toll of fractures can be substantial, affecting not only the individuals themselves but also their families and caregivers (Chang et al., 2016).

Researchers have actively pursued strategies for fall prevention to minimise the adverse consequences of slip-related falls (Aprigliano et al., 2017; Parijat and Lockhart, 2008; Chou et al., 2011; Moyer et al., 2009; Parijat et al., 2008; Nazifi et al., 2017; Marigold and Patla, 2002). Wearable devices, such as orthosis, hold significant promise in reducing the incidence and thus the associated economic and social burdens of falls (Karlsson et al., 2013; Monaco et al., 2017). Additionally, the deployment of real-time wearable assistive technologies offers users a heightened sense of safety, potentially mitigating the fear of falling and enabling individuals to maintain their daily activities more confidently (Monaco et al., 2017; Trkov et al., 2017; Mioskowska et al., 2020). This, in turn, can lead to reduced post-fall rehabilitation needs, resulting in cost-effective outcomes and a more efficient allocation of human resources.

Monaco et al. (2017) introduced the Active Pelvis Orthosis (APO), a wearable device designed to aid balance during slip perturbations. The APO detected perturbations by comparing real-time hip angles with those predicted by adaptive oscillators, while maintaining transparency during normal walking. Trkov et al. (2017) developed the Robotic Knee Assistive Device (ROKAD), offering knee torque during slips. It compared knee angular velocities and torques between normal walking and slips, using impedance and torque feedback control. Mioskowska et al. (2020) introduced a knee assistive device preventing slip-related falls by actively extending the trailing leg’s knee. The study focused on actuation strategy, not Loss of Balance (LoB) detection, demonstrating rapid knee extension in benchtop tests.

Despite these promising research results, there is still a need for a more comprehensive understanding of the mechanics of falls and the role of specific lower limb joints in generating effective responses. This understanding is essential for informing the design of wearable devices aimed at preventing slips and falls. These devices primarily focus on detecting instances of LoB and generating timely actuations to restore a biomechanically stable position, enabling individuals to regain a steady walking gait following a slip-like perturbation. Proposed strategies include varying detection times, ranging from 30 to 300 ms after the slip, and actuation times, which can range from 200 to 350 ms after the slip, as documented in (Monaco et al., 2017; Mioskowska et al., 2020; Trkov et al., 2017).

However, analysing the biomechanical response to slip events is vital in comprehending the role and importance of individual muscles and joints engaged in the biomechanical process, gathering knowledge to set device specifications. Consequently, a wealth of essential knowledge can be gathered to inform the design and development of these devices. Traditionally, the literature has predominantly focused on studying the sagittal kinematic movements of the hip, knee, and ankle joints in order to prevent falls following slip perturbations. However, frontal plane movements, especially involving ankle and hip dynamics, are very relevant in stabilizing gait and averting falls. Thus, it is needed a broader research framework encompassing parameters from frontal plane (Kim et al., 2019; Aprigliano et al., 2016). Other research has explored changes in spatiotemporal variables from the onset of the slip to the completion of the recovery process (Aprigliano et al., 2015a; Hirata et al., 2021). Electromyographic (EMG) data, including variables such as muscular synergies, latency periods, and peak magnitudes, are commonly analyzed to understand the muscles involved in slip recoveries and gain insights into the characteristic lower limb movements during slip recoveries (Qu et al., 2012; Chambers and Cham, 2007; Yoo et al., 2019). Some studies have also investigated the influence of different variables, such as intensity and gait speed, to induce variability in slip events and examine their respective biomechanical responses (Aprigliano et al., 2015a; Klemetti et al., 2014). However, there is still a need for a comprehensive analysis that integrates and complements all these types of data. This analysis should encompass factors related to human characteristics, such as muscle latency, as well as system specifications including Range of Motion (RoM), Torque, and rotations per minute (rpm). Additionally, it should take into account various environmental conditions, such as surface inclination (Chang et al., 2016). This is particularly important given the divergent or lack of findings in the scientific literature regarding muscle latency, RoM, Torque, and rpm. Clarifying these relationships and incorporating diverse environmental scenarios can contribute to advancing the current state of the art.

Considering these limitations, we intend to analyse slip-induced perturbations acquiring experimental kinematic, spatiotemporal, and EMG data through well-defined protocols encompassing various critical conditions. This is the initial step in enabling biomechanical analysis and its interpretation, and it will allow the comprehension of the specific roles of lower limb joints and muscles in slip recovery. Additionally, the influence of various variables such as gait speed, surface inclination, slipping foot, and perturbation intensity will be examined to determine their impact on slip recoveries. We also aim to rank the importance of lower limb joints in slip recovery, providing valuable information for informed decision-making on actuation strategies. Ultimately, we intend to establish target specifications for the development of a wearable robotic device to effectively prevent slip-related falls, facilitating the selection of appropriate mechanical components to mimic the relevant kinematic and spatiotemporal variables involved in human slip recovery.

## 2 Slip event temporal progression

When slips initiate, 60–120 ms after the Heel-Strike (HS) (Beschorner et al., 2013; Lockhart, 2013), they disrupt the subject’s Center of Mass (CoM) relative to the Base of Support (BoS), resulting in a backward LoB. In response, sensory systems detect this deviation and transmit the information to the motor control areas of the Central Nervous System (CNS) through afferent nerves. The CNS interprets the sensory input and generates efferent signals that selectively activate skeletal muscles to counteract the LoB by appropriately contracting and maintaining the body’s position within the BoS. The coordinated activation of lower limb muscles helps to resist foot displacement and facilitates slip recovery (Lockhart, 2013).

**FIGURE 1:**
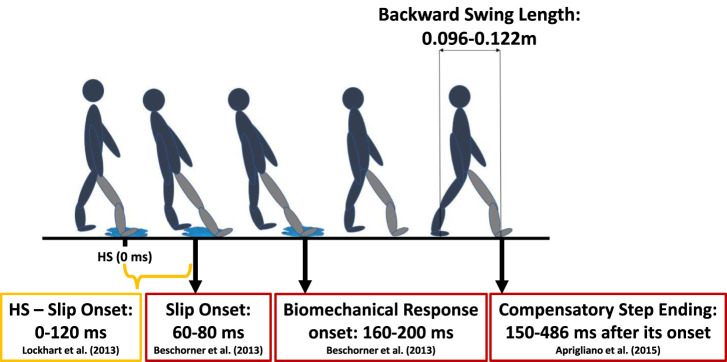
Overview of the temporal progression associated with the slip event, spanning from the moment of HS to the restoration of balance through the natural biomechanical response (Aprigliano et al., 2015b; Beschorner et al., 2013; Lockhart, 2013). Slipping leg is presented in grey.

Beschorner et al. (2013) investigated variables with higher deviations during slip responses compared to normal human gait. Notably, they found that the knee of the slipping leg exhibited dominant extensor movement, reaching maximum angular velocity 111 ms after slip onset. This was followed by changes in ipsilateral hip velocity (149 ms), plantarflexion of the leading ankle angle (156 ms), and hip flexion (170–200 ms). The trailing knee’s response occurred at 170 ms, and trailing hip extension happened at 200 ms. While these kinematic changes coincide with the muscle latency period (160–200 ms), it is suggested that these biomechanical deviations are primarily a result of the slip perturbation itself rather than the biomechanical response. Nonetheless, these movements play a crucial role in triggering the initial postural response (Beschorner et al., 2013). Notably, leading foot somatosensorial and knee velocity changes are among the first variables to shift, potentially critical in initiating the initial postural response. Moreover, joint movements that become apparent 200 ms after the onset of a slip, especially the responses of the trailing leg, are recognized as the earliest postural reactions to slips — a biomechanical phenomenon well-documented in the literature (Beschorner et al., 2013; Moyer et al., 2009; Kim et al., 2019; Hirata et al., 2021; Bhatt et al., 2006; Huntley et al., 2019; Lee et al., 2019; Yoo et al., 2019). Additionally, Aprigliano et al. (2015b) found that the compensatory step, defined as the time elapsed between perturbation onset and the HS of the trailing foot, increased with higher perturbation intensity. Martelli et al. (2017) confirmed these findings when examining compensatory step length and its flight time. In their research, the compensatory step was notably longer among the elderly (Young: 0.410 ± 0.01 s, Elderly: 0.480 ± 0.02 s). In summary, Figure 1 provides an overview of the timings associated with the slip event, according to the current literature.

## 3 Methods

### 3.1 Data acquisition

After contextualizing the literature, our study implements two experimental protocols to analyze slip-induced perturbations and gather comprehensive kinematic, spatiotemporal, and EMG data. These protocols investigate lower limb joint and muscle roles in slip recovery under varied conditions like gait speed, surface inclination, slipping foot, and perturbation intensity. Detailed equipment descriptions are in Section 3.1.2. The second protocol, while identical in nature to protocol 1, uses fewer equipment and includes an inertial sensor in the rope for efficient perturbation intensity measurement. We measured various physical quantities related to the subjects’ movements along three distinct axes: Anteroposterior (AP), Vertical (V), and Mediolateral (ML).

#### 3.1.1 Participants

For the first protocol, a total of 11 able-bodied subjects (six males and five females) were included in the study. The participants had a mean age of 24.55 ± 2.15 years, a mean height of 1.70 ± 0.09 m, and a mean weight of 63.25 ± 7.11 kg. In the second protocol, four subjects were selected, presenting a mean age of 24.55 ± 2.15 years, a mean height of 1.76 ± 0.05 m, and a mean weight of 72.00 ± 5.00 kg. Eligible subjects were required to meet the following criteria: i) be 18 years of age or older; and ii) have a body mass below 135 kg. Subjects with any disease or deficit that could affect locomotion or who had recently undergone surgical procedures that might impact mobility were excluded from the study. Prior to participating in the study, all individuals provided written and informed consent. The research adhered to the ethical guidelines defined by the University of Minho Ethics Committee (CEICVS 063/2021), which aligns with the principles outlined in the declaration of Helsinki and the Oviedo Convention. As part of the study protocol, each participant underwent a qualitative assessment of their preferred foot, using the Waterloo Footedness Questionnaire (Elias et al., 1998), contributing to a comprehensive understanding of locomotor asymmetries and potential influences on slip-induced movements. All participants in both protocols exhibited right dominance.

#### 3.1.2 Equipment

First Protocol: A comprehensive range of sensors was employed to capture various aspects of motion and physiological data. Participants were equipped with the Xsens MVN Awinda system, which comprised 17 Inertial Measurement Units (IMUs) placed at specific body landmarks to monitor changes in motion kinematic variables during both normal walking and slip perturbations. These landmarks included the head, sternum, pelvis, shoulders, upper arms, forearms, hands, upper legs, lower legs, and feet. Data were collected at a rate of 60 Hz. After sensor placement, participants underwent N-Pose calibration to ensure accurate measurements. Reflexive markers were also affixed to various body landmarks, including the head, sternum, midtrunk, shoulders, elbows, wrists, hips, knees, heels, and feet. These markers were tracked at 120 Hz using the Optitrack V120 Trio camera bar. Any shiny surfaces on the participants’ clothing were minimized to reduce noise captured by the Optitrack cameras.

**FIGURE 2:**
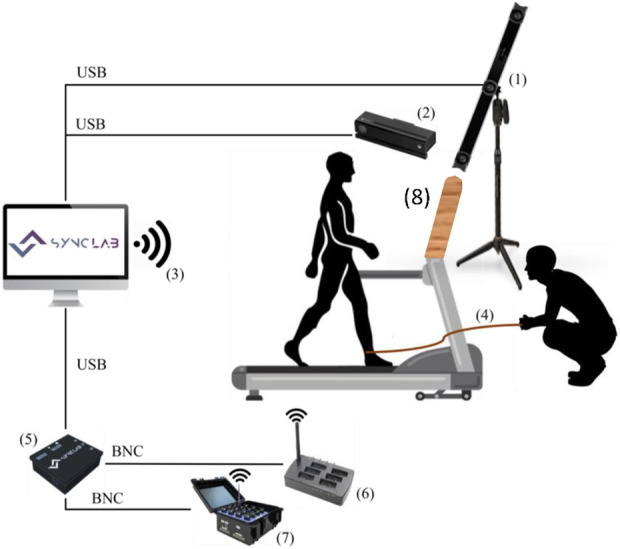
Experimental setup used for slip-like perturbations data collection. 1) Optitrack V120 Trio cameras. 2) Kinect v2.0 camera. 3) Wireless communication between the computer running the RespiBAN app and Shimmer systems. 4) Rope attached to the participant’s ankle, which is pulled by the operator to induce the perturbation. 5) Sync Box to deliver electronic pulses. 6) Xsens Awinda station, which establishes wireless communication with the Xsens IMUs. 7) Delsys Trigno Workstation, which establishes wireless communication with the Delsys sensors. 8) Barrier between participant and operator.

Delsys Trigno wearable sensors were placed on specific lower-body muscles, namely the Rectus Femoris (RF), Biceps Femoris (BF), Tibialis Anterior (TA), and Gastrocnemius Lateralis (GL) of both legs. These sensors recorded EMG data at approximately 1,111 Hz. Three Maximum Voluntary Contraction (MVC) trials were performed for each muscle to facilitate the subsequent normalisation of EMG envelopes. The RespiBAN system was worn on the upper trunk, between the sternum and Xiphoid process, to collect respiration data. The Shimmer GSR device was placed on the dominant forearm with electrodes positioned on the middle fingers and index to get information on galvanic skin response and heart frequency rate. Data from these devices were collected at rates of 1,000 Hz and 100.21 Hz, respectively. Additionally, the Kinect camera captured video recordings at a rate of 30 frames per second for video support in event labeling. Figure 2 provides an overview of the experimental trial used for data collection.

During the trials, subjects wore a safety harness system to prevent falls in the event of an irreversible LoB. This system involved a vest connected to a ceiling structure using a rope. The length of the rope was adjusted to maintain a minimum distance of 15 cm between the knees and the treadmill belt when the subject was suspended (Okubo et al., 2019). This adjustment was made by instructing subjects to lift their feet, thereby applying their full body weight to the harness system. To achieve synchronized data acquisition from all the sensors, the Sync Lab Desktop Application for Windows OS developed by our team, was employed. Trigger signals were sent by electronic pulses or via wireless.

Second protocol used Xsens and Delsys sensors exclusively. For the Xsens IMUs, participants were equipped with sensors only on the lower limbs. Additionally, an Xsens IMU was placed on the rope to allow more precise detection of perturbations and to study the influence of perturbation intensity by collecting rope accelerations. Similar to the first protocol, data from these sensors were collected at a frequency of 60 Hz, and participants underwent the N-Pose calibration of the system. Delsys Trigno wearable sensors were positioned on the same muscles as in the first protocol.

#### 3.1.3 Experimental protocols

During both experimental protocols, subjects were instructed to walk on a treadmill while managing unexpected slip-like perturbations. To prevent any prior bias on their biomechanical response, participants were not informed about the specific protocol. Prior to the slip perturbation trials, a familiarization trial was conducted, where subjects walked on the treadmill without any induced slip-like perturbations while wearing the entire sensor setup. To simulate real-world slip perturbations, a trained operator discreetly pulled a concealed rope tied to the subjects’ ankle at either a HS or Toe-Off (TO) event. Throughout all the trials, the rope remained attached to one of the subject’s feet, keeping the participants unaware of when a perturbation would occur. Moreover, the subjects were not able to see the operator to minimize potential bias. In the first protocol, each participant underwent eight trials, experiencing all possible combinations of perturbed gait events (HS or TO), slipping leg (right or left leg), and treadmill inclination (0° and 10°). The second protocol focused on perturbations induced only at HS, with 0° inclination applied to both legs, and at the same speeds defined in the first protocol. In both protocols, each subject experienced a total of 24 perturbations (12 at HS and TO; 12 per leg; 12 per inclination). Only perturbations at HS were considered for this study. Each trial consisted of six sub-trials, wherein subjects walked at three different speeds: 1.8 km/h, 5.4 km/h, and a normalized speed calculated based on the subject’s leg length (Equation 1). The selection of slowest and fastest gait speeds followed established literature guidelines (Klemetti et al., 2014; Kyrdalen et al., 2019; Quach et al., 2011). For each velocity, one sub-trial involved a slip-like perturbation, while the other sub-trial served as a non-perturbation control. The normalized speed (v) for each subject was calculated using the dynamic similarity principle expressed by Equation 1 (Martelli et al., 2013):

$$v=Fr\cdot \sqrt{g}\cdot L,$$ (1)

where Fr represents the Froude number (0.15), g is the acceleration due to gravity (9.81 m/ $s^{2}$ ), and L is the leg length measured from the prominence of the greater trochanter external surface to the lateral malleolus. The sequence of these sub-trials was randomized to ensure the unpredictability of the perturbations. In trials where perturbations were introduced, the operator applied three perturbations at random moments during the trial. Non-perturbation trials had an average duration of 30 s, while perturbation trials varied in duration, typically ranging from 30 s to 1 min. A new perturbation was consistently introduced 20–30 s after the previous one, ensuring that the subject had ample time to fully recover their balance before the next perturbation.

### 3.2 Data processing

The collected EMG data were normalized using Delsys Analysis software with the corresponding MVC information. The Optitrack reflexive markers were labelled using Motive software, while excluding markers that were obstructed during the trial. Additionally, the frames captured by the Kinect camera were aligned using Adobe Premiere Software to generate a video for each trial.

To ensure consistency, the sampling frequency of the data was adjusted to 60 Hz (Xsens), excluding the Kinect v2.0. Subsequently, the data from each sensor were organized into tables, where each column represented a specific feature extracted from the sensors. All sensor data were aligned and standardized to have an equal number of samples for each trial.

Furthermore, the data were labelled based on specific events of interest, including the start and end of each sub-trial, perturbation onset, end of perturbation, start and end of the biomechanical response, and the recovery of steady walking. To perform the labelling, Djv software was utilized for the first protocol, as it enabled identification of Kinect frame numbers. Throughout the labeling process, it was noted that specific perturbations did not significantly destabilize the subject and, consequently, were excluded from the dataset. The timestamps provided by the Sync Lab Desktop App were correlated with the table timestamps to accurately mark the events in the trial data. In the case of the second protocol, the end of perturbation was determined by analyzing the minimum value of the AP Ground Reaction Force (GRF) following perturbation onset. The onset of the perturbation was determined using the accelerometer signal from the IMU placed on the rope.

### 3.3 GRF and torque estimation

The Customizable Toolbox for Musculoskeletal simulation (CusTOM) toolbox was used to estimate the GRF as well as the torques involved in each of the joints, from the Xsens inertial data, during the two protocols (Muller et al., 2019; Crowninshield, 1978). This toolbox facilitates inverse dynamics-based musculoskeletal analysis by leveraging the collected inertial data (Crowninshield, 1978), after appropriate calibration the musculoskeletal model of the specific subject involved in the trial.

### 3.4 Clustering and perturbation intensity

In order to analyze the intensity of each perturbation, the k-means clustering procedure was employed (Hastie et al., 2009). The clustering approach provides a quantitative framework for distinguishing between different levels of perturbation intensity, allowing for a more detailed analysis of the biomechanical responses and other variables of interest associated with each intensity cluster. The intensity of each perturbation was measured using the sum vector magnitude of the accelerations recorded by the IMU placed in the rope during protocol 2. These intensity values were then classified into three distinct groups: soft, intermediate, and severe. The clustering was based on the squared Euclidean distance metric. To maintain analytical consistency, we exclusively considered trials from protocol 2, wherein perturbations occurred within 400 ms before the nearest HS. This duration of 400 ms was selected to ensure an adequate amount of data for studying the interaction effect of all conditions. To further refine the categorization of perturbation intensity, we explored various methods and criteria, ultimately opting for three levels to achieve a balanced distribution among perturbations: those up to 20 g, those up to 40 g, and those exceeding 40 g. We believe this approach provides a robust framework for categorizing perturbation intensity and enables a nuanced examination of biomechanical responses across different levels of perturbations.

### 3.5 Study dependent variables

A comprehensive assessment of the biomechanical response to slip perturbations was conducted by carefully selecting relevant Dependent Variables (DVs), namely kinematic, spatiotemporal and EMG variables, in order to provide a comprehensive understanding of slip-induced biomechanical changes. We aim to identify the role and significance of each muscle and joint involved in the biomechanical process and define future devices’ specifications. Additionally, hip movements in the frontal plane, such as adduction/abduction angles, were included, which expanded the scope of the investigation. The selected variables, categorized by their respective groups, are presented in Table 1.

**TABLE 1:** General approach and DVs analyzed in the included articles.

Kinematic variables (angles mean values)	Spatiotemporal variables (mean values)	EMG variables
Right hip - frontal plane	CoM velocity - AP (CoM _ x)	Latency
Left hip - frontal plane	CoM velocity - ML (CoM _ y)	Excitatory power response
Right hip - sagittal plane	CoM velocity - V (CoM _ z)	Inhibitory power response
Left hip - sagittal plane	Distance between both feet (AP & ML)	
Right knee - sagittal plane	Distance between both feet (AP, ML & V)	
Left knee - sagittal plane	Distance between CoM and Right foot	
Right ankle - sagittal plane	Distance between CoM and Left foot	
Left ankle - sagittal plane		

Parameters related to the CoM enable the investigation of subject balance behavior during steady walking, perturbation, and biomechanical response (Vlutters et al., 2016). Therefore, CoM velocities in AP, ML, and V directions were considered. The distances between the CoM and both feet, as well as the distances between the right and left feet, were also incorporated, considering both two-dimensional and three-dimensional measurements.

The selected variables included latency periods, EMG inhibitory and excitatory responses. Following the approach of Marigold and Patla (2002) and Qu et al. (2012), distinct activation thresholds were established for excitatory and inhibitory responses. The excitatory response corresponded to muscular activity above the mean of steady walking plus two standard deviations, while inhibitory responses were associated with activity below the mean minus two standard deviations of steady walking. It is important to note that the mean and standard deviation values were calculated solely during steady walking to ensure their independence from the number of perturbations induced in each sub-trial. Additionally, these calculations were performed for each speed and subject.

Muscular activation latency was determined by measuring the interval between HS and the onset of muscle activity. HS was identified using the contact points provided by Xsens software, while muscle activity onset was defined as the time when the EMG signal first deviated above or below the activation threshold for at least 30 ms (Marigold and Patla, 2002). Furthermore, EMG power was calculated by integrating the EMG signal above or below the activation threshold for excitatory and inhibitory responses, respectively. All these variables were extracted for each detected HS, and the data were subsequently labelled according to perturbation and non-perturbation conditions.

The average values of kinematic and spatiotemporal variables were computed. To calculate these average values, the data were initially segmented sequentially based on the labelling of each frame, distinguishing between steady walking, slip-like perturbation, and biomechanical response periods. Subsequently, means were calculated for each segmented period.

### 3.6 Statistical analysis

Analysis of Variance (ANOVA) is employed to investigate the influence of multiple Independent Variables (IVs), including gait speed, surface inclination, slipping foot, and perturbation intensity, on slip recoveries. This statistical analysis will help us discern the significance of each IV under study and EMG data, as well as their collective impact on the outcomes of interest. First, the assumptions of ANOVA, namely, multicollinearity analysis, sample independence, and multivariate normality (Finch, 2005), were examined to ensure the validity and reliability of the results. Considering the number of observations in the datasets obtained from the two protocols (n = 659 for the first dataset and n = 96 for the second dataset), assumptions regarding data homogeneity and linear relationship between the IVs and DVs (Table 1) were not checked. ANOVA is known to be robust against violations of these assumptions for group sizes larger than thirty. Outliers were not excluded due to the datasets size (Allen and Bennett, 2008).

In the first dataset (larger than 300 samples), skewness (<2) and kurtosis (<4) values were used to test normality, while the Kolmogorov-Smirnov test was employed for the second protocol dataset (Mishra et al., 2019). In the second protocol, when the *p*-value is greater than 0.05, the null hypothesis is accepted, indicating that the data is normally distributed. To investigate this assumption in ANOVA, the data were initially split according to the labelling of the IVs included in the analysis.

A Pearson correlation test was performed to assess multicollinearity between the DVs. A Pearson coefficient with an absolute value higher than 0.9 indicates a strong correlation between variables. In such cases, one of the correlated variables was selected, while the other was discarded as it provided approximately the same information.

A significant *p*-value (<0.05) indicated differences between population means. To determine specific differences between means, a Tukey-B *post hoc* test was performed, which accommodates pairwise comparisons between groups of different sizes (Allen and Bennett, 2008). Effect size was also included to quantify the magnitude of differences between groups (eta squared - $\eta^{2}$ ) (Richardson, 2011). The slip-like perturbation variable was included in all ANOVA tests to assess its effect across different conditions, while the slipping foot variable differentiated the roles of the slipping and trailing legs. Two-way and three three-way ANOVA analyses were conducted, combining perturbation and slipping foot with other IVs (speed, inclination, and intensity) and EMG data, followed by Tukey-B *post hoc* analysis.

### 3.7 Variable ranking procedures

To rank the different DVs and understand their quantitative influence on the biomechanical response to slip perturbations, two distinct approaches were pursued. Firstly, the effect size of the ANOVA models was considered in the statistical analysis, utilizing the partial $\eta^{2}$ value to quantify the influence of a specific variable in generating significant differences between groups. $\eta^{2}$ values were calculated for all the analyzed ANOVA models (Richardson, 2011), since this value naturally changes when the ANOVA model is altered. Consequently, the ranking of variables based on this value provides insights only for a specific ANOVA model. Variables with a partial $\eta^{2}$ value lower than 0.01 indicate a low variation in means due to the interaction of the analyzed DVs. Values between 0.01 and 0.06, and values higher than 0.14 correspond to situations of medium and large effect sizes, respectively (Richardson, 2011).

Regarding the second approach, variable ranking was also conducted using Feature Selection Methods (FSMs), commonly employed in Machine Learning techniques (Roffo et al., 2017): i) Infinite Latent Feature Selection (ILFS); ii) Infinite Feature Selection (InfFS); iii) Extended Correlation-based Feature Selection (ECFS); iv) Minimum-Redundancy Maximum-Relevancy (MRMR); v) Relief-F; vi) Multi-Cluster Feature Selection (MCFS); vii) norm regularized discriminative feature selection for unsupervised learning (UDFS); viii) Local Learning-based Clustering Feature Selection (LLCFS); ix) Correlation-based Feature Selection (CFS); and x) FSASL. These methods assess the differences between variables when comparing two distinct situations or classes. After the FSM analysis, the obtained rankings for each variable were normalized on a scale of 0–1. The normalized ranks were then summed for each variable, enabling the comprehensive ranking of all DVs. In similarity-based FSMs, variables with higher sum values were prioritized, whereas in dissimilarity-based methods, DVs were ranked in ascending order.

### 3.8 Target specifications definition

The definition of the required RoM for each joint of the lower limbs was examined using the angle values obtained from Xsens. For each of the three gait phases (steady walking, perturbation, and biomechanical response) considering both the slipping and trailing legs, maximum and minimum values were extracted. Similarly to the procedure followed for torque values, outliers in RoM were removed.

The detection $\left(\Delta t_{d}\right)$ and actuation $\left(\Delta t_{a}\right)$ times based on scientific literature (Trkov et al., 2017; Mioskowska et al., 2020; Monaco et al., 2017) provided insights into the timings involved from the initiation of a slip event until the restoration of steady walking. By combining the $\Delta t_{d}$ (detection time) and $\Delta t_{a}$ (actuation time) with the Range of Motion (RoM) values, the required number of rotations per minute (rpm) for the robotic wearable device motors could be defined, depending on the specific joint requiring actuation, according to the following Equation 2. Rpm quantifies the rate at which these joints rotate or move during dynamic activities such as walking or recovering from slips.

$$rpm=\frac{{\text{ROM}}_{\text{max}}\cdot 0.60}{\Delta t_{a}}$$ (2)

## 4 Results and discussion

### 4.1 Clustering and classification of perturbation intensity

The results of the clustering and classification procedure are summarized in Table 2 and include the maximum and minimum values of the sum vector magnitude of the accelerations for each intensity cluster, as well as the number of perturbations in each cluster. The soft intensity cluster comprised 19 perturbations, with a maximum value of 212.83 m/ $s^{2}$ and a minimum value of 50.99 m/ $s^{2}$ . The intermediate intensity cluster consisted of 16 perturbations, ranging from a maximum of 346.67 m/ $s^{2}$ to a minimum of 235.32 m/ $s^{2}$ . Finally, the severe intensity cluster comprised 12 perturbations, with a maximum value of 599.40 m/ $s^{2}$ and a minimum value of 393.35 m/ $s^{2}$ .

**TABLE 2:** Perturbations’ intensity clustering properties obtained using matlab k-means algorithm.

Cluster	Maximum value (m/s^2^)	Minimum value (m/s^2^)	No. of perturbations
Soft	212.83	50.99	19
Intermediate	346.67	235.32	16
Severe	599.40	393.35	12

### 4.2 Statistical analysis outcomes

#### 4.2.1 Perturbation vs. foot

**FIGURE 3:**
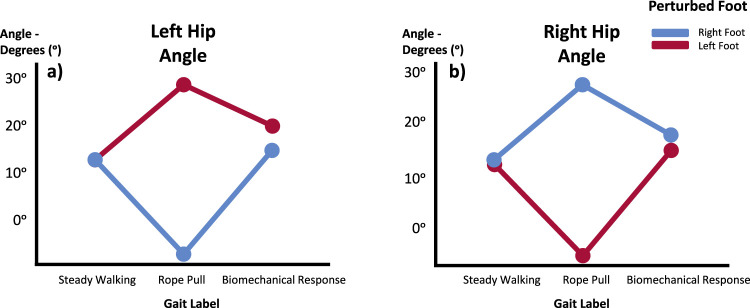
Hip angle means in the Sagittal Plane for Left **(A)** and Right **(B)** during Steady Walking, Rope Pull, and Biomechanical Response. Analysis considers the foot perturbed. Increasing angles indicate flexion, while decreasing angles indicate extension movements.

Statistically significant differences (*p* < 0.005) were observed in the variables associated with joint movement in the sagittal and frontal planes, specifically in the hip, when considering the interaction between perturbation and slipping foot. This suggests notable variations in the average values of these variables. Figure 3 depicts the evolutionary graphs demonstrating the average values of right and left hip angles before, during and after the perturbation. Furthermore, *post hoc* analysis was conducted to identify the specific areas where these average differences occur. By analyzing the interaction between the perturbation and slipping foot IVs, valuable insights can be gained regarding the distinct roles played by the slipping and trailing legs.

Both the left and right hip angles exhibit similar patterns. The hip of the slipping leg demonstrates an increase in average values during the rope pull phase (indicative of flexion movement) compared to steady walking. Subsequently, during the biomechanical response phase, the average values decrease, suggesting a predominant hip extension of the slipping leg. Conversely, when analyzing the hip in relation to the perturbation on the contralateral side, a reflexive behavior is observed in the plots. Initially, during the rope pull phase, there is a decrease in the average values, indicating a dominant extensor moment. This is followed by an increase in the average values, signifying a flexion movement of the joint. In both cases, the average values during steady walking phases closely resemble those obtained after the biomechanical response. The Tukey-B *post hoc* analysis identified significant differences between the three gait phases in the average values of the right hip and between the rope pull phase and biomechanical response phases in the situation of the left hip.

The findings suggest that the extension of the slipping leg’s hip counteracted the destabilizing effect by bringing the slipping foot closer to the CoM, while the flexion movement of the trailing leg’s hip brought both feet closer together, increasing stability. These findings are concordant with the scientific literature (Aprigliano et al., 2015a; Beschorner et al., 2013).

Similar findings were observed in the knees’ response. The average values during steady walking and the biomechanical response phases were highly similar, indicating an effective biomechanical response that helps prevent falls. The ipsilateral knee exhibited knee extension during the rope pull phase, while knee flexion movement was dominant during the biomechanical response phase. As for the contralateral knee, it primarily displayed knee flexion during the perturbation phase, followed by knee extension after the biomechanical response. In the case of the contralateral knee, significant differences were found between the rope pull phase and biomechanical response labels for the right knee, and among all three gait phases for the left knee, according to the Tukey-B *post hoc* analysis.

Both ankles also exhibited statistically significant movement patterns. Analyzing the behavior of the perturbed ankle, dorsiflexion movement was observed during the rope pull phase, while plantarflexion was dominant during the biomechanical response. In the contralateral position, both the right and left ankles displayed different movement patterns when comparing steady walking to the rope pull phase. However, during the transition from the rope pull phase to the biomechanical response phases, both ankles exhibited a dominant plantarflexion movement. The Tukey-B *post hoc* analysis revealed significant differences among all three gait phases for both ankles on both sides.

Significant differences were observed in the movements of the hips in the frontal plane. In the case of the contralateral hip, a notable strategy was observed, wherein both feet were brought closer together through hip adduction. As for the ipsilateral hip during the biomechanical response phase, different movements were observed for both hips. The right hip did not exhibit a statistically significant difference in mean values, while the left hip demonstrated an abduction movement. The *post hoc* tests revealed significant differences between steady walking and rope pull phases for the mean values of the left hip in the frontal plane. For the right hip, differences were primarily observed in the rope pull label when comparing it with both steady walking and the biomechanical response phases. Additionally, the distance between both feet increased during the rope pull phase compared to steady walking phase, and then decreased during the biomechanical response phase. The *post hoc* test indicated significant differences among all three gait phases in terms of the defined distances.

#### 4.2.2 Perturbation vs. foot vs. inclination

**FIGURE 4:**
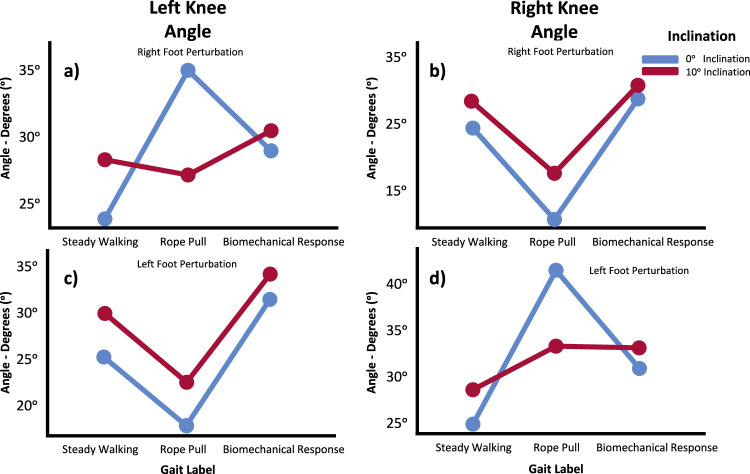
Knee angle means in the Sagittal Plane for Left **(A, C)** and Right **(B, D)** during Steady Walking, Rope Pull, and Biomechanical Response. Analysis considers interaction effects of perturbation, foot perturbed, and inclination. Increasing angles indicate flexion, while decreasing angles indicate extension movements.

When examining the interaction effect of perturbation, slipping foot, and inclination, statistical significance was observed only in both knees. The Tukey-B *post hoc* analysis revealed more pronounced differences in mean values during the transition from rope pull phase to the biomechanical response phase in the right knee, and differences among all three gait phases in the left knee. In the case of the ipsilateral knee, there were no significant differences in the evolution of the plots when comparing both joints. However, at a 10° inclination, the mean values were higher. Comparing the contralateral side of both knees for both inclinations, it appears that inclination influences the response of the knee. In trials with 0° of inclination, the knee flexes during the rope pull phase and then extends during the biomechanical response phase. However, in trials with 10° of inclination, the evolution differs between the right and left knees. For the right knee on the contralateral side, it is slightly flexed during the perturbation and slightly extended during the biomechanical response phase. Conversely, the left knee exhibits a reflexed behavior in comparison. Figure 4 depicts these outcomes.

#### 4.2.3 Perturbation vs. foot vs. speed

When examining the interaction effect between perturbation, slipping foot, and speed, all joint angles were found to be statistically significant except for the left hip means in the sagittal plane. Tukey-B *post hoc* tests revealed significant differences in the left hip means in the sagittal plane when considering the three speed labels (1.8 km/h, 5.4 km/h, and self-selected speed). Analyzing both hips average values, it can be observed that speed does not influence the extension and flexion movements in this joint, whether it is ipsilateral or contralateral, or under different gait phases. Increased speed resulted in higher mean values regardless of the hip’s positioning or gait condition.

**FIGURE 5:**
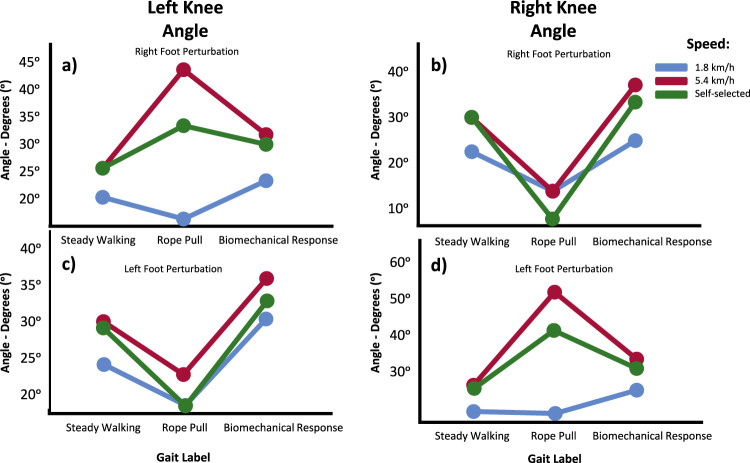
Knee angle means in the Sagittal Plane for Left **(A, C)** and Right **(B, D)** during Steady Walking, Rope Pull, and Biomechanical Response. Analysis accounts for interaction effect between perturbation, foot perturbed and gait speed. Increasing angles represent flexion, while decreasing angles represent extension movements.

Regarding the angles of the knees, the Tukey-B *post hoc* speed analysis indicated differences between all gait phases for both knees. The knee graphs generally support the main results obtained in the previously described models regarding the dominant movements observed during gait phases (Figure 5). However, the contralateral knee exhibited a reflexed behavior at lower velocities. When considering the effect of speed on the evolution of these graphs, it can be observed that higher velocities corresponded to higher mean values regardless of whether the knee was ipsilateral or contralateral, or under different gait conditions.

In terms of the ankle joints, there was no consistent relationship between speed and mean values when analyzing the ipsilateral ankle. However, in the case of the contralateral ankles, consistent results were found for both the left and right ankles. In this scenario, speed not only influenced the subjects’ response to the perturbation but also affected their biomechanical response phase. At self-selected and 5.4 km/h speeds, the contralateral ankle exhibited the same biomechanical response. However, at lower velocities (1.8 km/h), the response of the contralateral ankle showed a reflexed pattern: during the perturbation, this joint was plantarflexed, and during the biomechanical response phase, it mainly exhibited dorsiflexion. Tukey-B *post hoc* analysis considering the speed effect revealed significant differences between the three gait phases for both the right and left ankles.

Tukey-B *post hoc* tests also revealed that the difference in frontal movements of both hips is primarily caused by the perturbation variable rather than the interaction between perturbation and speed. Regarding the *post hoc* analysis of speed, significant differences were found only for the right hip in situations involving higher speeds. When examining hip angles in the frontal plane, the ipsilateral hips did not show consistent patterns when comparing both feet. On the other hand, the contralateral hips supported the previously discussed conclusions for higher velocities, while at 1.8 km/h, the contralateral hip exhibited a reflexed behavior in the frontal plane.

In terms of the distance between both feet, Tukey-B *post hoc* tests demonstrated statistical significance for both perturbation and speed variables. As mentioned earlier, the rope pull led to a greater distance between both feet, which was subsequently reduced during the biomechanical response phase. Higher speeds resulted in a larger distance between both feet compared to lower velocities. Although not statistically significant for the interaction between perturbation and speed, it is expected that the CoM velocity in the AP direction would have higher values for higher velocities, regardless the considered foot.

Higher speeds resulted in increased flexion of the hip and knee joints, regardless of whether they were ipsilateral or contralateral to the perturbation. The ipsilateral ankle showed a consistent response similar to the previous analysis. However, the contralateral ankle exhibited different responses depending on speed. At higher velocities, the response was similar to the previous analysis, while at lower velocities, the ankle showed a reflexed response with plantarflexion during the perturbation and dorsiflexion during the biomechanical response. This difference can be attributed to the timing of the slipping leg’s HS coinciding with an earlier stage of the propulsive phase of the contralateral limb. The contralateral knee also showed differential responses, with extensor dominance observed at lower velocities. The inconsistency in hip angles in the frontal plane between both feet can be explained by the right-dominant nature of the subjects. Higher speeds were also associated with greater distance between the feet, while lower speeds resulted in shorter periods of double stance, making it easier to induce more noticeable perturbations. At a 10° inclination, the knees showed increased flexion across all gait phases. The response of the contralateral knee was less variable in trials with a 10° inclination. More intense perturbations led to a greater distance between the feet and increased flexion in both knees during the perturbation. These results align with existing literature that highlights a larger distance between the CoM and BoS for more intense perturbations (Aprigliano et al., 2015a). Stronger perturbations also resulted in higher RoM values for the right hip and left knee, indicating a compensatory response to counteract the destabilizing effect of the perturbation.

#### 4.2.4 Perturbation vs. foot vs. intensity

In the analysis of the interaction effect between perturbation, slipping foot, and intensity using the data collected in the second protocol, only the distance between the CoM and the left foot showed statistical significance (*p*-value < 0.05). Although some DVs were not determined to be significant, Tukey-B *post hoc* tests revealed significant differences in certain DVs depending on the intensity of the perturbation. Regarding the frontal plane movements of the hips, Tukey-B *post hoc* tests determined that for severe perturbations, the right hip exhibited statistically significant differences in both soft and severe perturbations, while the left hip did not show statistically significant differences depending on the perturbation intensity. In the ipsilateral hips, there was an increase in the mean during the rope pull phase, followed by a reduction during the biomechanical response phase, indicating hip adduction. For more intense perturbations, the increase in means during the rope pull phase was more pronounced, and in these cases, the biomechanical response phase of the right hip showed a higher RoM.

Regarding the sagittal plane of the same joint, Tukey-B *post hoc* tests identified significant differences in the means of the left hip during severe perturbations. Upon examining the graphs, it can be concluded that the biomechanical response phase of both hips on the contralateral side is not modulated by the intensity of the perturbation. However, the ipsilateral hips, both right and left, exhibited higher flexion movements during the rope pull phase for more intense perturbations. The right hip also showed a greater RoM in its biomechanical response phase to more intense perturbations.

In analyzing the behavior of the knee means during perturbations, Tukey-B *post hoc* tests did not reveal statistical significance for the right knee across the three perturbation intensities. However, the left knee showed a statistical difference for both severe and soft perturbations. Analyzing the joint’s behavior, it can be observed that when the knee is placed ipsilaterally to the perturbation, there are only slight differences depending on the perturbation intensity. On the other hand, when the knee is on the contralateral side, the biomechanical response phase appears to be intensity-dependent for the right knee: the more intense the perturbation, the more pronounced the reduction in mean values, and the biomechanical response phase shows a higher RoM.

**FIGURE 6:**
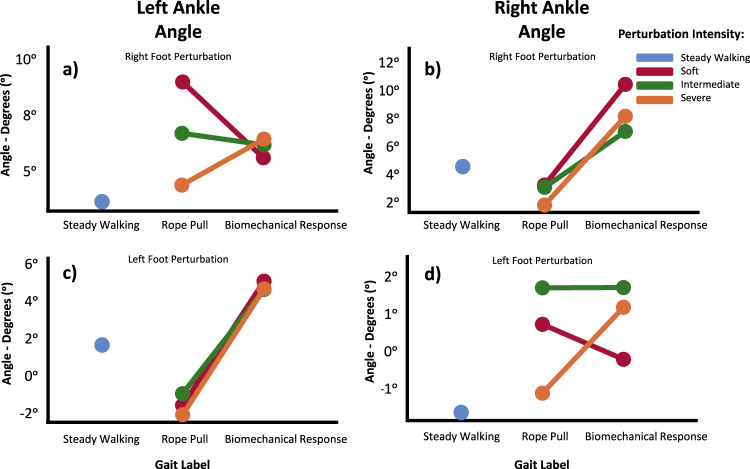
Ankle angle means in the Sagittal Plane for Left **(A, C)** and Right **(B, D)** during Steady Walking, Rope Pull, and Biomechanical Response phases. Analysis accounts for interaction effects of perturbation, foot perturbed, and perturbation intensity. Increasing angles represent flexion, while decreasing angles represent extension movements.

Regarding the behavior of the left and right ankles (Figure 6), when the perturbation is delivered ipsilaterally to these joints, there are no significant differences in their evolution, as they are characterized by a plantarflexion movement. However, when the perturbation is delivered in the contralateral side, the results obtained do not exhibit a clear pattern.

Finally, in terms of the distance between the feet, Tukey-B *post hoc* tests determined statistically significant differences for intermediate and severe perturbations. As expected, for both the left and right feet, more intense perturbations induced a greater distance between them.

#### 4.2.5 Perturbation vs. EMG data

In examining the interaction effect of perturbation and slipping foot on EMG variables, the following statistical significances were discovered: i) right and left BF excitatory responses; ii) right and left RF inhibitory responses; iii) right RF excitatory response; iv) right and left GL excitatory responses; and v) right and left GL inhibitory responses. Consistent results were observed for BF, as both the right and left BF excitatory responses showed statistical significance, while both inhibitory responses were not statistically significant. Although both the right and left BF excitatory responses increased during perturbations on the right and left sides, the perturbations on the right side resulted in a greater increase in the excitatory response of both right and left BF.

Regarding RF, both inhibitory mean variations were statistically significant, while only the right RF excitatory response showed statistical significance in the ANOVA analysis. Perturbations delivered to the left foot led to stronger inhibitory responses in both the right and left RF, while perturbations on the right foot resulted in evident excitatory responses in the right RF.

**FIGURE 7:**
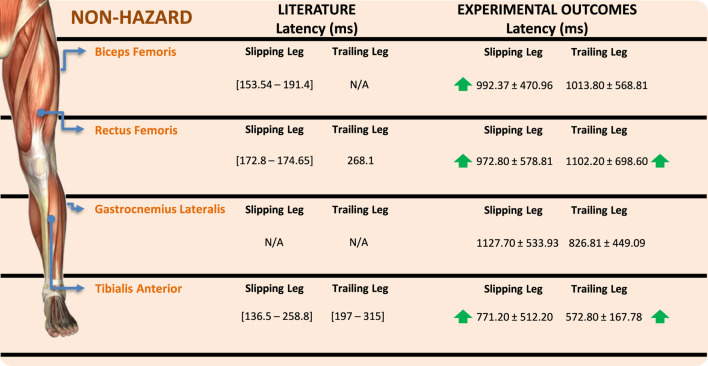
Muscle latency periods comparison between experimental data and scientific literature.

For GL, both excitatory and inhibitory responses were found to be statistically significant for both the right and left sides. Similar to the results observed for BF, perturbations on the right foot induced a higher increase in GL muscle power means. Additionally, both inhibitory responses were more pronounced during perturbations delivered to the right limb. Despite the same tendency, the excitatory and inhibitory responses of the right GL were associated with higher means compared to the left GL. On the other hand, no statistically significant differences were found for the excitatory responses of the TA muscle, and no inhibitory responses were observed from this muscle. Regarding the analyzed latency periods for the four muscles included in this study, the ANOVA did not find statistically significant differences in the interaction effect between perturbation label and slipping foot. The latency periods are shown in Figure 7, compared with the scientific literature. Only non-hazardous latency periods were included in this comparison, as no subjects experienced falls during the experimental trials.

The interaction effect between perturbation and slipping foot was found to be statistically significant for the excitatory responses of the right and left BF muscles. The increased excitatory responses align with the extension strategy of the slipping leg’s hip joint and the flexion strategy of both knees. Surprisingly, right perturbations resulted in more powerful responses in both the right and left BF muscles, indicating a potential dominance effect that warrants the inclusion of left-footed subjects in future protocols. Inhibitory and excitatory responses of the GL muscles were also determined to be statistically significant. These muscles contribute to ankle plantarflexion and knee flexion, which are key kinematic strategies observed in the biomechanical response phase to slip perturbations. The results confirm the importance of GL in stabilizing the ankle joint and promoting contact between the foot and floor to increase the BoS. Inhibitory responses of the RF muscles were significant for both the right and left limbs, indicating inhibition of knee extension during the biomechanical response phase. This aligns with the dominant knee flexion movement observed in the slipping leg’s response. Excitatory responses of the right RF muscle were also significant, possibly influenced by subject dominance and the role of knee extension as a secondary strategy for steady walking resumption after slip-like perturbations, as supported by previous literature (Chambers and Cham, 2007).

During the conducted EMG data analysis, the interaction effect between perturbation label and slipping foot did not yield statistically significant differences in muscular latency periods. The latency periods obtained in the experimental analysis were found to be greater than those reported in scientific literature (Chambers and Cham, 2007; Nazifi et al., 2017; Marigold and Patla, 2002). This disparity may be due to the time gap between the HS and the onset of induced perturbations, as they were not simultaneous in most cases. Additionally, the use of a rope to provoke perturbations, as opposed to controlled platforms used in literature, may alter the muscular response due to the application of external force.

### 4.3 Variable ranking

Upon analyzing the results obtained from the partial $\eta^{2}$ values (Table 3), it is evident that in the case of the two-way ANOVA for perturbation versus slipping foot, the majority of the obtained values are higher compared to the other ANOVA models. This indicates that the interaction between these IVs is primarily responsible for the significant variation observed in the means of the DVs under investigation. Among these variables, the mean joint angle values in the sagittal plane exhibit the most significant influence due to the interaction effect of perturbation and slipping foot. Specifically, the hip joint means display the most pronounced changes in mean values, followed by the knee and ankle joints, respectively. Regarding the remaining models, in the analysis of the interaction effect between perturbation and gait speed, the mean values of both the knee and ankle joints in the sagittal plane stand out with higher values compared to the others. The variable ranking was further determined through the FSM as presented in Table 4.

**TABLE 3:** Partial $\eta^{2}$ values per ANOVA model for all the DVs. Small, medium, and large effect sizes are, respectively, shading at purple, orange and green.

DV	Perturbation vs. foot	Perturbation vs. foot vs. inclination	Perturbation vs. foot vs. speed	Perturbation vs. foot vs. intensity
Right hip frontal AVG	0.0765	0.0046	0.0284	0.040
Left hip frontal AVG	0.0409	0.0051	0.0172	0.0011
Right hip sagittal AVG	0.6081	0.0049	0.0186	0.0006
Left hip sagittal AVG	0.6123	0.0070	0.0103	0.0048
Right knee sagittal AVG	0.2773	0.0144	0.0629	0.0136
Left knee sagittal AVG	0.1115	0.0256	0.0451	0.0333
Right ankle sagittal AVG	0.0595	0.0025	0.0602	0.0120
Left ankle sagittal AVG	0.0441	0.0067	0.0522	0.0097
CoM × velocity	0.0016	0.0000	0.0012	0.0000
CoM y velocity	0.0014	0.0004	0.0005	0.0000
CoM z velocity	0.0006	0.0004	0.0016	0.0000
3D foot distance	0.0035	0.0040	0.0261	0.0040
Distance CoM - right foot	0.0000	0.0000	0.0022	0.0433
Distance CoM - left foot	0.0005	0.0000	0.0037	0.0322

**TABLE 4:** FSM results.

Foot distance	Left ankle	Distance CoM – right foot	Right hip frontal	Right hip	Right knee	COM velocity *z* direction	Distance CoM – left foot	Left knee	Right ankle	COM velocity *y* direction	Left hip	Left hip frontal	COM velocity *x* direction
8.54	8.04	7.38	6.96	6.96	6.88	6.79	6.54	6.42	6.29	6.29	6.08	5.71	5.46

Examining the FSM results, among all the considered DVs, the distance between the feet exhibits the highest sum value, indicating its ability to better differentiate between all gait phases. As for the DVs related to joint angle means, the left ankle ranks first. The DVs associated with the right hip also occupy prominent positions, specifically the fourth and fifth positions. Distinguishing between the joints of the left and right legs, for the right leg, the descending order of the sum values for these variables is hip, knee, and ankle. Conversely, for the left leg, the descending order is reversed, with the ankle ranking first, followed by the knee and the hip, respectively.

$\eta^{2}$ and FSM revealed that the interaction between perturbation and slipping foot had the greatest influence on the variation of the DVs. Joint angle averages in the sagittal plane exhibited the highest variations, with the hip joints showing the most pronounced alterations, followed by the knee and ankle joints. The results suggest that the hip joint plays a more active role in the biomechanical response to slip-like perturbations compared to the ankle joint, which is consistent with existing literature (Qu et al., 2012). The FSM analysis revealed that the distance between both feet was the variable with the greatest ability to distinguish all gait phases. The joints on the right side, except for the left ankle, ranked lower compared to their counterparts on the left side. Once again, this discrepancy may be attributed to the right-handedness of the subjects and the absence of consideration for the perturbation side in the analysis.

### 4.4 Torque, RPM and RoM

**FIGURE 8:**
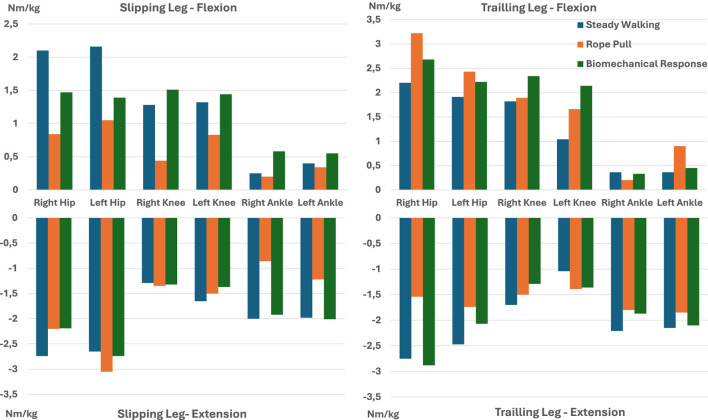
Joint torques estimated using CusToM toolbox.

The minimum and maximum torque values for each lower limb joint, estimated using the CusToM toolbox, are summarized in Figure 8. These values provide insights into the torque patterns during different gait phases for slipping and trailing legs. It can be observed that the maximum and minimum torques are primarily associated with the rope pull and biomechanical response labels under certain circumstances. This finding highlights the importance of incorporating slip-induced procedures and considering their outcomes to design purpose-oriented devices that account for the specificities of slip-like disturbance scenarios.

**FIGURE 9:**
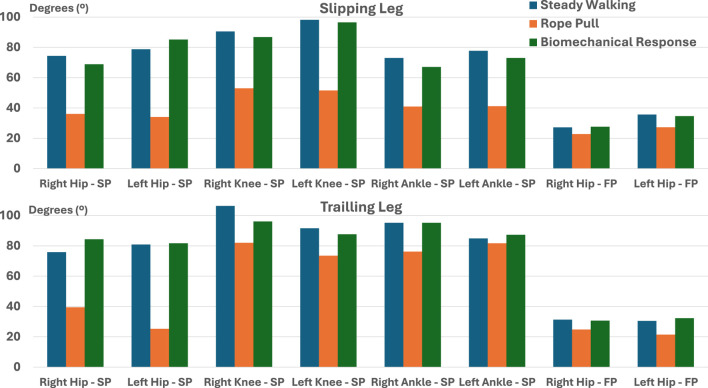
Lower limb’s joints RoM, during steady walking, rope pull, and biomechanical response obtained by experimental data analysis (SP, Sagittal Plane; FP, Frontal Plane).

**FIGURE 10:**
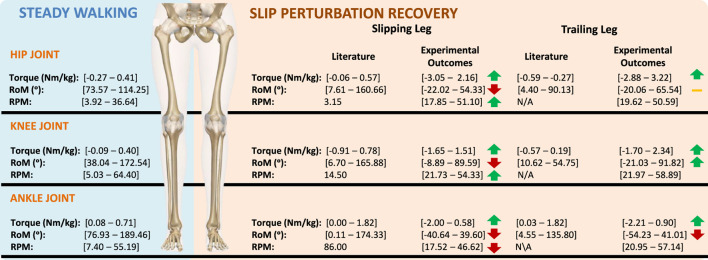
Torque, RoM and rpm comparison between literature results and experimental data outcomes.

Figure 9 depicts these values for each of the three gait phases. Similar to the findings in the torque analysis, extreme RoM values are generally associated with rope pull and biomechanical response phases. This further confirms the destabilizing effect of slip-like perturbations. Finally, Table 5 presents the range of rpm values obtained by considering $\Delta t_{a}$ . The knee and ankle joints exhibit higher rpm values compared to the hip joints. Figure 10 provides a comparison between the torque, RoM, and rpm obtained from the experimental data, along with the corresponding metrics reported in the scientific literature. RoM values for the hip and knee align with existing literature, indicating flexion/extension movements of approximately 140° and 150°, respectively (Nancy Hamilton et al., 2011; Grimmer et al., 2020). However, the ankle angles exceed the reported RoM values in scientific literature for all three gait phases, although they are smaller than those presented in (Kim et al., 2019). Consequently, rpm values are naturally affected. Moreover, higher values can be attributed to IMU displacements caused by perturbations.

**TABLE 5:** Range of rpm values obtained considered the previously presented actuation times.

Joint	${rpm}_{minimum}$		${rpm}_{maximum}$	
	Slipping leg	Trailing leg	Slipping leg	Trailing leg
Right hip	17.85	20.24	44.62	50.59
Left hip	20.45	19.62	51.10	49.04
Right knee	21.73	25.52	54.33	63.80
Left knee	23.56	21.97	58.89	54.93
Right ankle	17.52	22.86	43.81	57.14
Left ankle	18.65	20.95	46.62	52.37

When comparing the torques obtained using the CusToM toolbox with literature on the development of wearable robotic devices for fall prevention, our values were found to be higher than those reported in studies such as Monaco et al. (2017), Mioskowska et al. (2020), and Trkov et al. (2017). Specifically, Monaco et al. (2017) defined a torque of 0.2 Nm/kg for hip actuation, while our work determined a maximum absolute torque value of 3.0 Nm/kg for the same joint. Regarding the knee joint, Mioskowska et al. (2020) and Trkov et al. (2017) reported torques of 20 Nm and 40 Nm, respectively. Although these values are not normalized by subjects’ weight, they might be lower than the 1.9 Nm/kg obtained in our study. This discrepancy in values can be attributed to errors associated with the CusToM toolbox and the method by which the perturbation was induced.

## 5 Conclusion

This study provides a comprehensive exploration of the biomechanical responses to slip-like perturbations across various gait conditions, shedding light on critical factors influencing stability and informing future advancements in fall prevention technology. Through detailed analysis, we uncovered distinct movement patterns in the lower limbs, highlighting the pivotal roles of the hip, knee, and ankle joints in responding to perturbations. Our findings underscore the significant impact of speed, inclination, and perturbation intensity on joint angles and responses, emphasizing their relevance in understanding gait stability dynamics. Notably, the extension of the slipping leg’s hip counteracted destabilization by bringing the slipping foot closer to the center of mass, while flexion movement of the trailing leg’s hip increased stability by bringing both feet closer together. One key revelation is the pronounced influence of the interaction between perturbation and the slipping foot on key DVs, particularly evident in the variability of joint angles, especially in the sagittal plane. Notably, the hip joints emerged as primary contributors to these variations, indicating their central role in mitigating slip-induced instability. These insights not only deepen our understanding of human biomechanics but also hold significant implications for the development of wearable robotic devices aimed at preventing slip-related falls. While our study achieved reasonable accuracy in estimating GRF and joint torques, disparities with existing literature underscore the ongoing need for refinement and standardization of experimental protocols. Despite these challenges, our investigation lays a solid foundation for future research endeavors, driving towards the establishment of a gold-standard protocol. However, the inclusion of force platforms would enhance precision. Ultimately, our findings pave the way for advancements in fall prevention technology, with the overarching goal of enhancing safety and wellbeing across diverse environments.

## Data Availability

The raw data supporting the conclusions of this article will be made available by the authors, without undue reservation.
